# Red forehead dot syndrome: a case report and review of literature

**DOI:** 10.3389/fpain.2026.1888803

**Published:** 2026-10-16

**Authors:** Walid Alesefir

**Affiliations:** Internal Medicine Department, Hail University, Hail, Saudi Arabia

**Keywords:** cranial autonomic symptoms, cutaneous manifestation, migraine, neurogenic inflammation, red forehead dot, trigeminal nerve

## Abstract

**Background:**

Migraine is a common neurological disorder with a broad spectrum of sensory and autonomic manifestations. Visible, recurrent, localized cutaneous changes temporally associated with migraine attacks are rare.

**Case presentation:**

I report a 41-year-old Pakistani man with an approximately 21-year history of recurrent bilateral frontal and supra-orbital headaches associated with photophobia, phonophobia, and nausea. For many years, his attacks have been accompanied by recurrent, well-demarcated bilateral erythematous forehead changes in a distribution corresponding approximately to the ophthalmic (V1) trigeminal territory. Because of the long duration of symptoms, he could not reliably determine whether migraine-type headaches preceded the cutaneous manifestations or whether both had been associated from the beginning. Intermittent cranial autonomic features occurred during some attacks but were not consistently present and did not invariably coincide with the skin discoloration. Neurological examination, laboratory evaluation, and brain MRI were unremarkable. Following his usual acute analgesic treatment, both headache and skin changes improved within minutes to a few hours.

**Conclusion:**

This case adds to the limited literature describing migraine-associated forehead erythema, previously termed “red forehead dot syndrome.” The reproducible temporal relationship between headache and cutaneous changes supports an association with the migraine attack, although the underlying mechanism remains uncertain. Trigeminovascular activation, neurogenic inflammation, autonomic dysfunction, and vasoactive neuropeptides such as calcitonin gene-related peptide (CGRP) are biologically plausible mechanisms. Because this presentation is rare, appropriate evaluation for secondary neurological, vascular, inflammatory, and dermatological causes remains essential.

## Introduction

Migraine is a common and disabling neurological disorder characterized by recurrent attacks of headache accompanied by symptoms such as nausea, photophobia, and phonophobia (1). Updated Global Burden of Disease (GBD) 2021 estimates indicate that approximately 1.2 billion people were living with migraine worldwide in 2021, with migraine accounting for substantially greater disability than tension-type headache (2).

Migraine is now understood as a complex neurobiological disorder involving cortical, brainstem, and trigeminovascular networks (3). Activation of trigeminal afferents and release of vasoactive neuropeptides, including CGRP, can contribute to vasodilation and neurogenic inflammatory signaling (4). Cranial autonomic symptoms such as lacrimation, conjunctival injection, nasal congestion, rhinorrhea, eyelid edema, and facial sweating, although classically associated with trigeminal autonomic cephalalgias, may also occur in migraine (5, 6).

Visible localized cutaneous changes accompanying migraine are much less commonly described. Previous reports have documented recurrent erythematous forehead dots, patches, ecchymotic changes, and linear markings occurring in temporal association with migraine attacks (7–11). In this report, I describe a patient with long-standing bilateral migraine-type headaches accompanied by reproducible bilateral forehead erythema and discuss the presentation in the context of the limited published literature.

## Case presentation

### Patient data and clinical timeline

A 41-year-old Pakistani man employed in a high-stress office job presented to the neurology clinic with an approximately 21-year history of recurrent headaches. His medical history was notable for lumbar disc disease. He had no known history of hypertension, diabetes mellitus, dyslipidemia, peripheral vascular disease, other significant cardiovascular disease, or autoimmune disorders. He was a non-smoker and had no known drug allergies. He reported a positive family history of migraine in first-degree relatives, but no family history of similar headache-associated skin lesions or other relevant dermatological disorders.

The bilateral forehead erythematous changes had also been present for many years and were repeatedly observed in association with headache attacks. Because of the long duration of his symptoms, the patient could not reliably recall whether migraine-type headaches initially preceded the cutaneous manifestations or whether the two phenomena had been associated from the beginning. Accordingly, a precise temporal sequence between the initial onset of the headache disorder and the first appearance of the forehead discoloration could not be established.

### Headache characteristics

The patient described recurrent bilateral pressure-like pain involving the frontal and supra-orbital regions. Attacks were associated with photophobia, phonophobia, and nausea. Headaches occurred approximately 2−3 times per month and lasted from several hours to occasionally days. The main reported triggers were psychological stress and sunlight exposure. He typically rested in a dark room and used an over-the-counter combination of acetaminophen, caffeine, and codeine.

Intermittent cranial autonomic features occurred during some headache attacks; however, they were not consistently present and did not invariably coincide with the forehead discoloration. Thus, the conventional autonomic features did not show a consistent temporal relationship with the cutaneous manifestation.

### Cutaneous manifestation and treatment response

A characteristic feature of the attacks was the appearance of well-demarcated erythematous changes over the bilateral forehead, corresponding approximately to the ophthalmic (V1) distribution of the trigeminal nerve and broadly mirroring the bilateral frontal and supra-orbital distribution of the headache (Figure 1). The lesions were described as non-palpable and non-pruritic. The patient reported a reproducible association between the skin changes and his headache episodes over many years.

**Figure 1:**
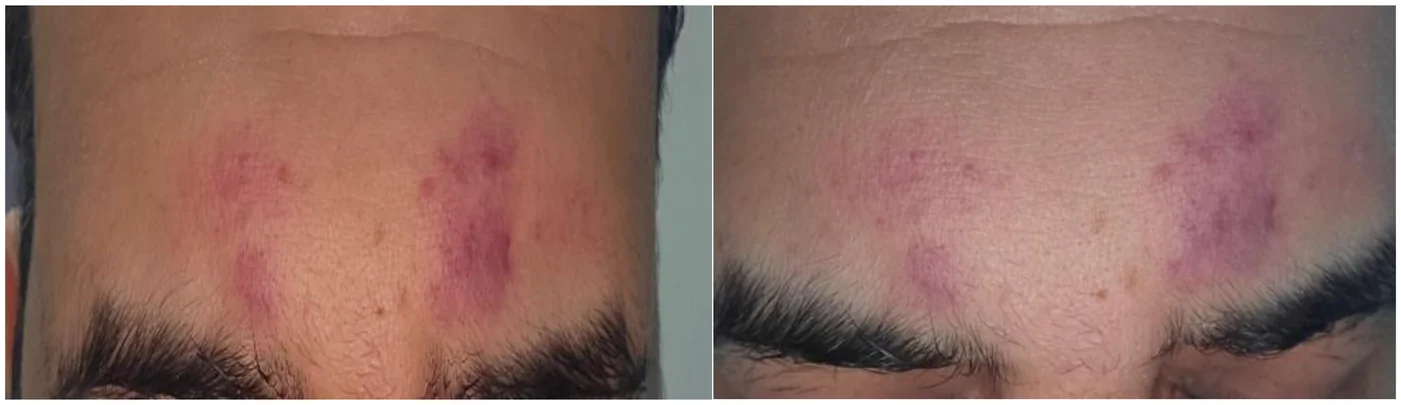
Clinical presentation of the migraine-associated forehead erythema. Photographs provided by the patient during a headache attack. Frontal view demonstrating bilateral well-demarcated erythematous forehead changes. Close-up view demonstrating homogeneous erythema and distinct borders.

Following his usual acute treatment with acetaminophen, caffeine, and codeine, improvement in both the headache and the associated forehead erythema occurred within minutes to a few hours. The cutaneous changes generally improved in parallel with the headache, supporting a close temporal association between pain improvement and resolution of the discoloration. At the clinic consultation, the patient was headache-free and did not have an active erythematous lesion; photographs obtained during attacks documented the characteristic bilateral forehead changes.

### Physical examination

The patient was afebrile and vital signs were within normal limits. His height was 187 cm, weight 88 kg, and body mass index 25.17 kg/m^2^. General and systemic examinations were unremarkable. Neurological examination, including mental status, cranial nerves, motor and sensory function, coordination, and gait, was normal, with no focal neurological deficits.

### Laboratory and imaging findings

An evaluation was undertaken to exclude clinically relevant secondary causes. Complete blood count, renal function tests, inflammatory markers (erythrocyte sedimentation rate and C-reactive protein), and vitamin B12 were within normal limits. Brain MRI without contrast showed preserved gray-white matter differentiation and no evidence of infarction, hemorrhage, mass lesion, hydrocephalus, mesial temporal sclerosis, cortical dysplasia, or focal brainstem or cerebellar lesion. Overall, the MRI was unremarkable (Figure 2).

**Figure 2:**
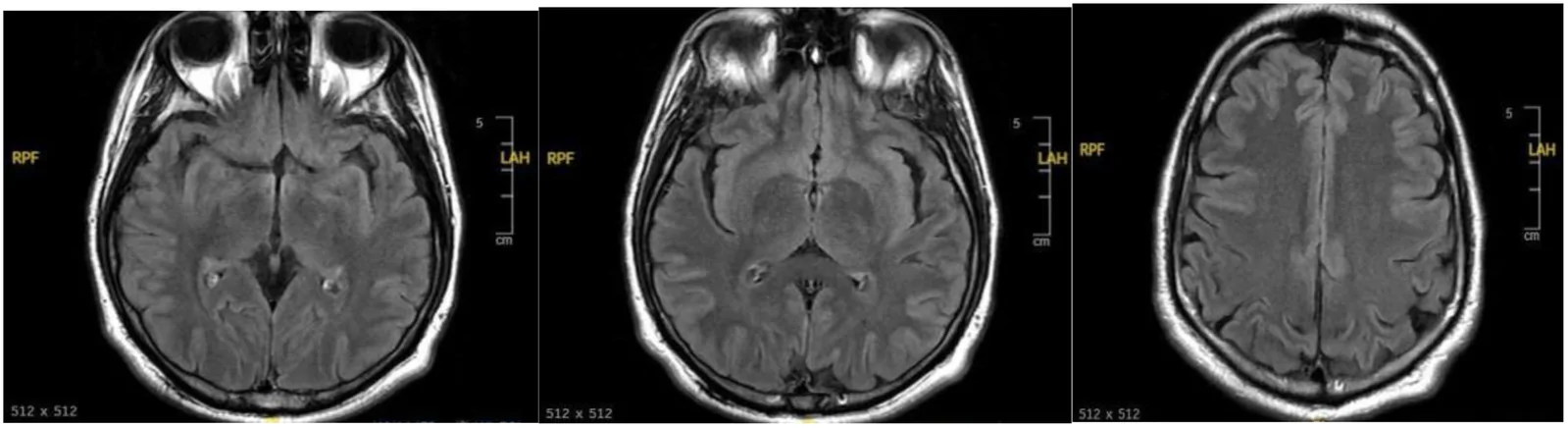
Normal brain MRI. Representative axial FLAIR and T2-weighted images demonstrating normal brain parenchyma without structural abnormality, infarction, hemorrhage, or mass lesion.

## Discussion

This case describes a middle-aged man with long-standing recurrent bilateral migraine-type headaches accompanied by reproducible bilateral forehead erythema. The cutaneous changes occurred in a distribution broadly corresponding to the V1 trigeminal territory and improved in parallel with headache relief. The patient also experienced intermittent cranial autonomic features, but these were inconsistent and did not invariably accompany the skin discoloration. This distinction suggests that the visible cutaneous phenomenon should not simply be assumed to represent a conventional cranial autonomic symptom, although overlapping trigeminal-autonomic and trigeminovascular mechanisms are plausible.

### Potential pathophysiological mechanisms

Activation of the trigeminovascular system is central to migraine biology. Trigeminal afferent activation can promote release of vasoactive neuropeptides, including CGRP, with downstream vascular and neurogenic inflammatory effects (4). Previous reports of migraine-associated forehead erythema have proposed extracerebral vasodilation, trigeminovascular activation, autonomic dysfunction, and neurogenic inflammation as possible mechanisms (7–11). Histopathological findings in one published case included perivascular dermatitis, extravasated erythrocytes, endothelial swelling, and fibrinoid material in vessel walls, supporting the possibility that vascular change may coexist with local inflammatory processes (10).

The availability of CGRP receptor antagonists (gepants) provides an additional opportunity to explore the proposed mechanism. In similar patients, prospectively documenting whether acute treatment with a gepant produces parallel improvement in both the migraine attack and the associated erythematous lesion could provide supportive functional evidence for CGRP-related trigeminovascular involvement. Such a therapeutic response, however, would remain hypothesis-generating and would not by itself establish causality.

### Clinical implications and diagnostic evaluation

Unusual cutaneous findings accompanying headache warrant careful assessment, particularly at first presentation. Given the rarity of migraine-associated forehead erythema, clinicians should consider and appropriately exclude secondary neurological, vascular, inflammatory, and dermatological causes according to the clinical context and the presence of red flags. Recognition of a stable, recurrent, and tightly headache-associated pattern should therefore complement, rather than replace, an appropriate diagnostic evaluation. After relevant alternative causes have been excluded, recognition of the phenomenon may help avoid unnecessary repeated investigations and may provide reassurance to the patient.

### Literature review

Published reports demonstrate heterogeneity in the morphology and timing of migraine-associated forehead skin changes (Table 1). Sethi et al. described the classic “red forehead dot syndrome,” including erythema extending toward the periorbital V1 distribution and, in some attacks, sweating (7). A later teaching neuroimage described a woman with a red forehead dot, eyelid ecchymosis, and V1-distribution redness, with normal MRI, magnetic resonance angiography, and venography (8). Berdouk and Khan reported a 28-year-old Pakistani man with recurrent migraine associated with extensive linear forehead markings that intensified with headache severity and resolved after the migraine (9). Durmaz and Demirkesen described a 36-year-old man with recurrent erythematous and telangiectatic forehead macules during severe migraine attacks; biopsy demonstrated perivascular inflammation and vascular changes (10). More recently, Muhammad et al. reported two Pakistani women with linear erythematous streaks or discoid erythematous patches associated with migraine attacks (11).

**Table 1:** summarizes these reports and the present case.

Study	Patient/ethnicity	Headache phenotype	Skin manifestation	Timing/course	Key findings/proposed mechanism
Sethi et al., 2007 (7)	40-year-old man; ethnicity not specified in current source	Episodic migraine	Red forehead dot; extension toward periorbital V1 region; sweating in some severe attacks	Appeared with attacks and resolved as attacks subsided	Trigeminovascular activation; extracerebral vasodilation mediated by vasoactive neuropeptides
Sethi et al., 2015 (8)	32-year-old woman; ethnicity not specified in current source	Episodic migraine	Red forehead dot with eyelid ecchymosis/redness in V1 distribution	Attack-associated	MRI/MRA/MRV normal; trigeminovascular/vasoactive-peptide mechanism proposed
Berdouk & Khan, 2018 (9)	28-year-old Pakistani man	Recurrent migraine	Multiple linear erythematous markings over forehead/nose, becoming darker with greater severity	Recurrent; resolved after migraine	MRI normal; mechanism uncertain; vascular/neurogenic association considered
Durmaz & Demirkesen, 2020 (10)	36-year-old man; ethnicity not stated	Recurrent severe migraine	Multiple ovoid/triangular erythematous and telangiectatic macules on forehead/hairline	Present during attacks; disappeared with migraine resolution	Biopsy: perivascular dermatitis, extravasated erythrocytes, endothelial swelling/fibrinoid vascular change; trigeminovascular and autonomic mechanisms discussed
Muhammad et al., 2024 (11)	Two Pakistani women	Migraine	Linear erythematous streaks in one patient; multiple discoid erythematous patches with edema in the other	Concurrent with migraine and resolved after improvement	Imaging normal; histopathology in one supported vascular change; vascular/neurogenic mechanisms proposed
Present case	41-year-old Pakistani man	Long-standing bilateral frontal/supra-orbital migraine-type headache; 2−3 attacks/month	Bilateral well-demarcated forehead erythema, approximately V1 distribution	Long-standing, reproducible association; pain and erythema improve within minutes to a few hours after usual acute therapy	MRI/laboratory evaluation unremarkable; intermittent autonomic features not consistently linked to erythema; trigeminovascular/CGRP-related mechanism hypothesized

### Limitations

This report has several limitations. First, it describes a single patient and therefore cannot establish the prevalence, specificity, or generalizability of this cutaneous manifestation. Second, the chronology of the initial headache and skin manifestations is limited by long-term patient recall; the patient could not reliably determine whether migraine-type attacks preceded the forehead changes or whether both had been associated from the beginning. Third, the temporal relationship between headache and cutaneous changes was based primarily on the patient's longitudinal history and photographs obtained during attacks, because no active lesion was present during the clinic assessment. Fourth, objective dermatological or vascular assessment of the lesion during an active attack was not performed. Finally, although trigeminovascular activation, autonomic dysfunction, neurogenic inflammation, and CGRP-mediated effects are biologically plausible, this case cannot establish a causal mechanism. Prospective documentation of future cases, including standardized photography, dermatological assessment, and treatment-response data, would help characterize this phenomenon more clearly.

## Conclusion

This case describes a rare, recurrent bilateral forehead erythema associated with long-standing bilateral migraine-type headaches. The close temporal relationship between headache and skin changes, together with improvement of both within minutes to a few hours after acute treatment, supports an association between the two phenomena. However, visible localized cutaneous changes remain uncommon in migraine, and the pathophysiological mechanism is not established. Trigeminovascular activation, neurogenic inflammation, autonomic pathways, and vasoactive neuropeptides such as CGRP remain plausible explanations. Recognition of this previously described phenomenon, often termed “red forehead dot syndrome,” may broaden awareness of the phenotypic spectrum of migraine, but appropriate evaluation to exclude relevant secondary neurological, vascular, inflammatory, and dermatological causes remains essential.

## Data Availability

The original contributions presented in the study are included in the article/Supplementary Material, further inquiries can be directed to the corresponding author.
